# Recurrence Rate and Risk Factors for the Recurrence of Ovarian Endometriosis after Laparoscopic Ovarian Cystectomy

**DOI:** 10.1155/2021/6679641

**Published:** 2021-01-25

**Authors:** Sornsittipong Wacharachawana, Paweena Phaliwong, Sinart Prommas, Buppa Smanchat, Kornkarn Bhamarapravatana, Komsun Suwannarurk

**Affiliations:** ^1^Department of Obstetrics and Gynecology, Bhumibol Adulyadej Hospital, Royal Thai Air Force, Thailand; ^2^Department of Preclinical Science, Faculty of Medicine, Thammasat University, Pathumthani, Thailand; ^3^Department of Obstetrics and Gynecology, Faculty of Medicine, Thammasat University, Pathumthani, Thailand

## Abstract

The aim of this study was to identify the recurrence rate and risk factors for the recurrence of ovarian endometriosis (OE) after laparoscopic cystectomy. This was a retrospective cross-sectional study. Subjects were OE cases who underwent laparoscopic ovarian cystectomy at Bhumibol Adulyadej Hospital (BAH). The period of this study was from January 2008 to December 2017. Ovarian histopathology and at least one-year follow-up after surgery were the prerequisite requirements. A total of 106 OE cases were included in the study. Subjects were classified into recurrence and nonrecurrence groups. It comprised of 24 and 82 cases, respectively. The mean age of the participant was 32.4 years old. The demographic characters of both groups were comparable. The recurrence rate after laparoscopic OE surgery in the present study was 22.6% (24/106). The average largest diameter of OE in the present study was 54.5 mm. Postoperative medical treatment (OR 3.15, 95% CI 1.14-8.74, *p* = 0.02) and postoperative pregnancy (OR 2.86, 95% CI 1.03-7.93, *p* = 0.04) were associated factors for recurrence decrement. The recurrence rate of OE after laparoscopic cystectomy was 22.6%. Postoperative medical treatment and postoperative pregnancy were a significant factor that lowered OE recurrence.

## 1. Introduction

Endometriosis is defined as the presence of an endometrial gland or stroma outside the uterus which induces a chronic inflammatory reaction [1]. It is found predominantly in women of reproductive age and is associated with pelvic pain and infertility [2]. The incidence of endometriosis ranges between 5 and 10 percent [3]. The ovary is the commonly affected organ with a prevalence of 17-44 percent [4].

Laparoscopic conservative surgery has been considered the gold standard treatment for ovarian endometriosis (OE). Excision of all visible endometriotic lesions, associated adhesions, and ovarian restoration were the objective of the surgery [5]. However, OE recurrence after conservative surgery is a more common concern. The recurrence rate of OE after ovarian cystectomy was 10-35% [6–10].

Recently, risk factors related to ovarian endometriosis recurrence, namely, lower age at the time of surgery, high revised American Society of Reproductive Medicine (rASRM) score, pre- or postoperative medical treatment, and postoperative pregnancy, were investigated [11]. It is interesting to see if any of those risk factors affect Thai OE patients.

This study is aimed at determining risk factors for the recurrence of OE after laparoscopic cystectomy.

## 2. Materials and Methods

This was a retrospective study done at Bhumibol Adulyadej Hospital (BAH), Bangkok, Thailand. The study was approved by the BAH ethics committee (IRB 77/63). Data were collected from medical records from infertile clinic patients of the Department of Obstetrics and Gynecology, BAH, between January 2008 and December 2017. Data from patients who underwent laparoscopic surgery with provisional diagnosis of OE during the study period were collected. Exclusion criteria were non-OE from pathologic reports, incomplete follow-up (follow-up of less than one full year), and incomplete medical data. All cases successfully underwent laparoscopic surgery by qualified laparoscopic trained gynecologists. Patient records of their follow-up visits of at least one full year postoperation were collected.

At the time of surgery, the creation of pneumoperitoneum was initiated by using Veress needle insertion with CO_2_ gas insufflation. A 10 mm trocar port was inserted at the umbilical area after the abdomen was fully insufflated in a classical manner; then, two 5 mm trocars were inserted at the left and right lower quadrant, respectively. All pelvic and abdominal organs were thoroughly visually examined before any further procedure. Ovarian cystectomy was performed. All relevant pathologic findings were noted. Lysis of any pelvic adhesion and pelvic endometriotic lesion was extirpated; then, bleeding was meticulously controlled and normal anatomy was restored.

The sample size was calculated based on the recurrence rate of OE after surgery at 45.1 percent [12]. Confident level, acceptable error, and *p* value were chosen at 95, 10 percent, and 0.05, respectively. Ninety-six cases were the least number of sample sizes that achieved statistical significance.

Data were extracted from hospital inpatient files and computerized documents. The recurrence of OE was defined as the presence of a persistent ovarian cyst that had a thin wall (>2 cm) with regular margins, a homogenous low echogenic fluid content with scattered internal echoes not resolved after several successive menstrual cycles [10]. General characteristic data of cases, age, weight, height, body mass index, menarche, marriage status, parity, history of previous endometriotic surgery, and symptoms before surgery were included. Clinical data included dysmenorrhea, pelvic pain, dyspareunia, infertility, ovarian affected, largest diameter of ovarian endometriosis, pre- and postoperative medical treatment, and associated disease; pelvic endometriosis, adenomyosis, myoma uteri, rASRM score, postoperative pregnancy, and recurrence time for at least one-year follow-up after surgery were collected.

### 2.1. Statistical Analysis

Statistical analysis was performed using the statistical package for social science (SPSS Inc., Chicago, IL, USA) for windows version 18. The chi-square test or Fisher's exact test was used to compare categorical data to evaluate the risk factors for OE recurrence. A *p* value less than 0.05 was considered statistically significant.

## 3. Results

**Figure 1:**
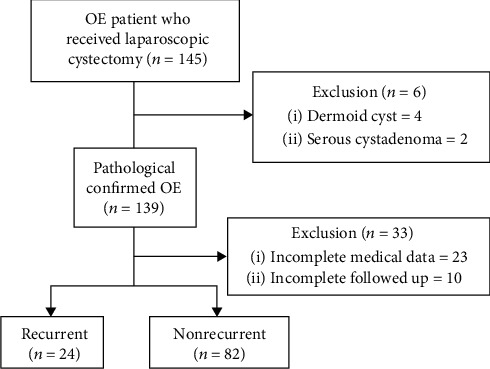
Sornsittipong Wacharachawana.

During the period of study, 106 subjects who had a minimum of one-year postoperative follow-up were recruited. A flow chart of the current study is shown in Figure 1.

Subjects were classified into recurrence and nonrecurrence groups of 24 and 82 cases, respectively. The recurrence rate in the present study was 22.6% (24/106). The mean postoperative period follow-up was 32.1 ± 5.8 months. The most leading symptom was dysmenorrhea (98/106). The average OE average diameter was 54.5 millimeters. Half of the cases (44/106) had bilaterality of the disease. Most cases had Category III and IV rASRM stages at 48.1 and 58.9 percent, respectively. Time interval to pregnancy occurred at an average of 16 ± 4.5 months after surgery. In those times, the physician gave either an oral contraceptive pill or depot medroxyprogesterone acetate (DMPA) to patients for 12 months. Pregnancy was usually seen one month after the discontinuation of the pills.

Demographic characters of all cases are presented in Table 1. The mean age of the patients was 34.0 ± 4.4 and 31.9 ± 5.6 years in the recurrence and nonrecurrence groups, respectively. The average basal metabolic index (BMI) of cases in the recurrence and nonrecurrence groups was 20.8 ± 4.1 and 21.3 ± 3.5 kg/m^2^, respectively. There was no statistical difference between recurrence and nonrecurrence groups in age, BMI, size of the cyst, multiparity, symptoms before surgery, and associated diseases.

Factors related to OE recurrence were analyzed by the chi-square test or Fisher's exact test and are represented in Table 2. The results of the analyses, age (≤30 years), BMI (>30 kg/m^2^), multiparous surgical history, symptoms before surgery, size of the cyst, bilaterality of the cyst, and associated disease were not significant risk factors of OE recurrence. The associated factors with OE recurrence were postoperative medical treatment (OR 3.15, 95% CI 1.14-8.74, *p* = 0.02) and postoperative pregnancy (OR 2.86, 95% CI 1.03-7.93, *p* = 0.04).

## 4. Discussion

This retrospective study was conducted in suburban referral hospitals in the northern part of Bangkok, Thailand. Most cases who attended an infertile clinic had an average age of 32.4 years old. Various studies of OE recurrent factors from Turkey, Korea, and China reported the mean age of their participants at 33.3, 34.2, and 33.2 years old, respectively [9, 10, 13]. The mean age of cases in the present study was slightly younger than the above-mentioned publication. Han et al. reported that the age of patients at the time of OE surgery had a correlation with the recurrence rate [9]. Han's work showed similar conclusions with Moini's and Yuan's works [6, 7, 9]. However, this current finding found no correlation that younger patients were correlated with the OE recurrence factor.

OE detected in the younger patients was related to an aggressive early onset nature of the disease [7]. Yuan's report indicated that patients with early OE onset should be concerned with a high recurrence rate. The high level of estrogen in young patients might induce recurrence [7, 9]. Most OE cases in current studies were not in the younger population. An estrogen level was not a part of this study. In our population, age was found to be an OE recurrent factor of OE, similar to other findings [8, 13].

Dysmenorrhea was a major symptom leading to OE diagnosis at BAH. Li's study from China reported that OE patients with dysmenorrhea had 1.7 times higher risk factors for OE recurrence [10]. In Li's work, dysmenorrhea level was subjectively determined for each case. Invasion of endometriosis and site of disease contributes to various inflammatory processes via the cytokine system. The aggressive disease that involved deep penetration and large area involvement proved itself difficult to be entirely eradicated by surgery. The remaining diseased tissue not removed by surgery was indicated to be associated with the recurrence of OE and its symptoms (dysmenorrhea) [10]. However, dysmenorrhea was not a major recurrence factor in this study. Our data could not retrospectively identify the level of dysmenorrhea as indicated in Li's recent work [10].

High rASRM stage (higher than stage III) referred to bilaterally large lesions of ovarian involvement and severe adhesions. A previous study from Japan, China, and Iran stated that the initial advance stage OE had a correlation to a higher rate of OE recurrence [6, 7, 12]. The recurrence rate of advance stage OE from our and Yuan's investigation was similar at 22.6% (24/106) and 20.5% (63/307), respectively [7]. Most cases in the current investigation were of advanced stages. The OE recurrence rates in stage III and IV patients showed no significantly different results.

Postoperative medical treatment in this study confirmed preventive factors for OE recurrence. Most cases were diagnosed at stage III (*n* = 51) and IV (*n* = 55) equally. Nearly half of the cases underwent postoperative hormonal treatment without statistical differences among different stages. Among cases who received postoperative hormonal treatment, 12.5 percent (6/48) were in OE recurrent status. The number was in lieu with Vercellini et al.'s meta-analysis work [14]. Vercellini et al. stated that 2.9-11.5 percent of cases who received postoperative hormonal treatment would develop recurrent disease [14].

The cases that did not receive postoperative hormonal treatment showed 3.15 times higher risk of OE recurrence than those undergoing postoperative hormonal treatment. Endometriosis was a benign hormonal dependent condition. The protective effect of OE recurrence was the suppression of ovarian function. Most hormonal treatments consisted of progestin. The protective effect of postoperative hormonal treatment in the present study supported Vercellini et al.'s work [14].

The postoperative pregnancy rate in the current study was 43.4% (46/106). Only thirteen percent (6/46) of cases who were pregnant after treatment developed recurrence OE events. The protective risk of pregnancy for OE recurrent was 2.86 times. Previous studies from Japan, Korea, and China stated that postoperative pregnancy could reduce the recurrence rate among OE cases that underwent laparoscopic surgery [7, 10, 12, 15].

The removal of endometriotic tissue was the key management in cases that further needed fertility function. Infertile women who received laparoscopic cystectomy showed improved fertility [16]. The success pregnancy rate in younger OE women was higher than in the older group [17]. The average age of OE patients who later got pregnant in our investigation was 32.4 years old. This result was in lieu with the result from Yuan's study with OE patients with an average age in later pregnancy at 34.2 years old [7].

The limitation of this study was the retrospective nature of the study. Selection bias and relative small number of patients who underwent laparoscopic cystectomy were unavoidable regarding patient characteristics. However, there was minimal missing data in the present study, and the numbers of the study cases were enough for the sample size to complete the statistical significance.

## 5. Conclusions

In conclusion, this study demonstrated that postoperative medical treatment and pregnancy were the protective factors associated with a lower recurrence rate of OE after laparoscopic cystectomy.

## Figures and Tables

**Table 1:** Characteristic of patients.

Factors	Total (*n* = 106)	Study (*n* = 24)	Control (*n* = 82)	*p* value
Age (years)^∗^	32.4 ± 5.4	34.0 ± 4.4	31.9 ± 5.6	0.13
Follow-up (month)^∗^	32.1 ± 5.8	36.7 ± 6.5	30.8 ± 4.9	0.88
BMI (kg/m^2^)^∗^	21.2 ± 3.6	20.8 ± 4.1	21.3 ± 3.5	0.74
Menarche^∗^	13.2 ± 1.3	13.6 ± 1.2	13.1 ± 1.3	0.64
Size of cyst (mm)^∗^	54.5 ± 27.5	53.0 ± 26.5	55.0 ± 27.9	0.94
Multiparous^∗∗^	29 (27.4)	4 (16.7)	23 (28.0)	0.30
History^∗∗^	6 (5.4)	2 (8.3)	4 (4.9)	0.62
Symptoms^∗∗^				
Dysmenorrhea	98 (92.5)	21 (87.5)	77 (93.9)	0.30
Pelvic pain	71 (67.0)	17 (70.8)	54 (65.9)	0.65
Dyspareunia	11 (10.4)	2 (8.3)	9 (11.0)	1.00
Infertility	61 (57.6)	16 (66.7)	45 (54.9)	0.30
Bilaterality of cyst^∗∗^	44 (41.5)	9 (37.5)	35 (42.7)	0.65
Associated disease^∗∗^				
Pelvic endometriosis	84 (79.3)	20 (83.3)	64 (78.0)	0.78
Adenomyosis	16 (15.1)	5 (20.8)	11 (13.4)	0.35
Myoma uteri	9 (8.5)	2 (8.3)	7 (8.5)	1.00
rASRM^∗∗^				
III	51 (48.1)	9 (37.5)	42 (51.2)	NA
IV	55 (51.9)	15 (62.5)	40 (48.8)	NA
Pre-op Rx^∗∗^	54 (50.9)	13 (54.2)	41 (50.0)	0.72
Post-op Rx^∗∗^	48 (45.3)	6 (25.0)	42 (51.2)	0.02
Post-op preg^∗∗^	46 (43.4)	6 (25.0)	40 (48.8)	0.04

Study: recurrence cases; control: nonrecurrence cases; ^∗^mean ± standard deviation (SD); ^∗∗^number (%); BMI: body mass index; history: surgical history; symptoms: symptom before surgery; rASRM: revised American Society of Reproductive Medicine; pre-op Rx: preoperative medical treatment; post-op Rx: postoperative medical treatment; post-op preg: postoperative pregnancy.

**Table 2:** Univariate analysis of factors related to OE recurrence.

Factors	Study	Control	OR	95% CI
*N* (%)	24 (22.6)	82 (77.4)	NA	14.50–30.70
Age ≤ 30 (years)	4 (16.7)	31 (37.8)	0.33	0.10–1.05
BMI > 30 (kg/m^2^)	1 (4.2)	3 (3.7)	1.15	0.11–11.54
Multiparous	4 (16.7)	23 (28.0)	0.51	0.16–1.66
History	2 (8.3)	4 (4.9)	1.77	0.30–10.33
Symptoms				
Dysmenorrhea	21 (87.5)	77 (94.0)	0.46	0.10–2.06
Pelvic pain	17 (70.8)	54 (65.9)	1.26	0.47–3.39
Dyspareunia	2 (8.3)	9 (11.0)	0.74	0.15–3.67
Infertility	16 (66.7)	45 (54.9)	1.64	0.63–4.27
Bilaterality of cyst	9 (37.5)	35 (42.7)	0.81	0.32–2.05
Size > 30 mm	20 (83.3)	72 (87.8)	0.69	0.20–2.45
Associated disease				
Pelvic endometriosis	20 (83.3)	64 (78.0)	1.41	0.43–4.64
Adenomyosis	5 (20.8)	11 (13.4)	1.70	0.53–5.48
Myoma uteri	2 (8.3)	7 (8.5)	0.95	0.19–5.03
rASRM				
III	9 (37.5)	42 (51.2)	NA	NA
IV	15 (62.5)	40 (48.8)	NA	NA
Pre-op Rx	13 (54.2)	41 (50.0)	0.85	0.44–2.11
Post-op Rx	6 (25.0)	42 (51.2)	3.15	1.14–8.74
Post-op preg	6 (25.0)	40 (48.8)	2.86	1.03–7.93

Study: recurrence cases; control: nonrecurrence cases; BMI: body mass index; history: surgical history; symptoms: symptom before surgery; rASRM: revised American Society of Reproductive Medicine; pre-op Rx: preoperative medical treatment; post-op Rx: postoperative medical treatment; post-op preg: postoperative pregnancy.

## Data Availability

The data used to support the findings of this study can be requested through the corresponding author.
